# Outpatient facility-based order variation in combined imaging

**DOI:** 10.1371/journal.pone.0224735

**Published:** 2019-11-14

**Authors:** Adam C. Powell, Yan Wang, Gary L. Smith, James W. Long, Uday U. Deshmukh, David P. Friedman, Christopher G. Roth, Baskaran Sundaram

**Affiliations:** 1 HealthHelp, Houston, TX, United States of America; 2 Humana Inc., Louisville, KY, United States of America; 3 Thomas Jefferson University, Philadelphia, PA, United States of America; Cleveland Clinic, UNITED STATES

## Abstract

**Objective:**

Combined computed tomography (CT) occurs when one anatomical area is simultaneously imaged both without and with contrast, or two overlapping anatomical areas are imaged concurrently. While this has been studied in a Traditional Medicare population, it has not been studied in other populations subject to prior authorization. This study explores between-facility variation in ordering and receiving orders to render combined CT in a mixed commercial and Medicare Advantage population.

**Methods:**

Orders for CT abdomen (without/with contrast), CT thorax (without/with contrast), and concurrent CT brain and sinus authorized by a prior authorization company from 2013–2017, pertaining to patients with commercial or Medicare Advantage health plans from one national insurer, were extracted. Orders were issued and rendered by both hospitals and nonhospitals. The analysis was performed separately for each anatomical area in two ways: orders were grouped by ordering facility, and by designated rendering facility. For each facility, the ratio of combined to total orders was calculated, and analysis of variance was used to determine whether there were significant differences in this rate by year. The association between health plan type and combined imaging rates was assessed.

**Results:**

Combined rates [ratio±standard deviation] for abdomen, thorax, and brain/sinus were 0.306±0.246, 0.089±0.142, and 0.002±0.01 respectively when the analysis was conducted according to ordering facility, and 0.311±0.178, 0.096±0.113, and 0.001±0.006 when the analysis was conducted according to designated rendering facility. Combined CT abdomen and CT thorax rates decreased monotonically from 2013 to 2017, decreases that were significant (P < .01) regardless of whether orders were grouped by ordering or rendering facility. Combined CT abdomen and CT thorax rates significantly differed between orders pertaining to people with commercial and Medicare Advantage plans.

**Discussion:**

Variability was greater when orders were grouped by ordering facility, rather than rendering facility. Health plan type may influence whether a patient receives combined CT.

## Introduction

Combined computed tomography (CT) is defined as the simultaneous imaging of one anatomical area both without and with contrast (e.g. without/with contrast CT of the abdomen, without/with contrast CT of the thorax), or two overlapping anatomical areas (e.g., noncontrast CT of the brain and sinus). When patients receive two CT procedures in combination, they inherently are exposed to more radiation than if they had received only one. When an order combines a CT acquired without contrast with a CT acquired with contrast, the patient is exposed to contrast. Although there are clinical situations in which combined imaging is beneficial, concerns over both radiation and contrast safety suggest that combined imaging should be used judiciously. [1, 2]

Combined imaging is a potential quality of care issue, as epidemiological studies have suggested the radiation dose resulting from even just two CT scans may increase a patient’s life risk of cancer. [1] Furthermore, concerns exist about the side effects of contrast agents. [2] Due to the potential risks associated with combined imaging, the Centers for Medicare & Medicaid Services (CMS) began examining combined imaging in 2007 as a part of the Hospital Outpatient Quality Reporting Program (HOQRP). [3] Under the HOQRP, hospitals are required to report data on a variety of outpatient quality measures, which are then shared with the public through the Hospital Compare website. Hospitals not complying with reporting requirements face a payment reduction of 2%. [4]

Although it is not always warranted, there are some occasions during which combined imaging is clinically warranted. For instance, the American College of Radiology (ACR) Appropriateness Criteria® for Chronic Liver Disease states that a CT of the abdomen conducted without and with contrast may be appropriate for screening and surveillance for hepatocellular carcinoma (HCC) among patients with no prior diagnosis of HCC, and as well as for surveillance among patients with a previous diagnosis of HCC. [5] Likewise, the ACR has released Appropriateness Criteria® stating that combined thoracic imaging may be appropriate for chylothorax treatment planning, as well as for the evaluation of chronic chest pain among patients with high probability of coronary artery disease. [6, 7] Similarly, while simultaneous CTs of the brain and sinus are often not needed due to the highly overlapping anatomy, such exams may be appropriate in situations involving comorbidities such as cancer, trauma, or orbital cellulitis. [8]

The three forms of combined imaging CMS started examining in 2007, combined CT of the abdomen, thorax, and brain/sinus, are substantial contributors to patient radiation exposure. Of the 29,000 future cases of cancer that researchers anticipate may be attributed to CT scans performed in 2007, the majority are expected to arise from scans of the abdomen and pelvis (14,000), chest (4,100), and head (4,000). [9] Thus, there may be a relatively large potential benefit to ensuring that future combined imaging of these areas is ordered judiciously.

As a result of the CMS HOQRP quality measures, combined imaging has been studied thoroughly in the context of claims analyses pertaining to patients with Traditional Medicare, a population which consists of older patients whose imaging orders are not subject to prior authorization. [4, 10–12] As older Americans are offered a choice between government-administered Traditional Medicare plans and privately administered Medicare Advantage plans, the two types of Medicare plans may differ in their patient populations in addition to their plan designs. Studies of Hospital Compare data related to these quality measures have been further limited, as they do not address imaging performed in nonhospital settings. [4, 11] Nonetheless, combined imaging is yet to be studied in the context of patients of diverse ages, utilizing data from a national private insurer that has implemented prior authorization. Likewise, the factors influencing the ordering of combined imaging have not been examined, as prior studies have been claims-based analyses which have only considered where combined imaging has been rendered.

The purpose of our study is to investigate the degree and nature of variation between facilities in ordering and receiving orders for outpatient combined imaging in a diverse population consisting of patients with both commercial and Medicare Advantage health plans offered by one national insurer, and to compare rates of combined imaging pertaining to people with commercial versus Medicare Advantage health plans. The health plans analyzed were subject to prior authorization for diagnostic imaging, further differentiating them from Traditional Medicare, which is not. Factors potentially associated with variation in combined imaging rates (year, site of service, urbanicity, health plan type, region) were examined in order to provide additional insights.

## Methods

### Data source and sample population

All authorized outpatient orders for abdominal, thoracic, brain, and sinus CT issued from 2013 to 2017, pertaining to patients with commercial and Medicare Advantage health plans from Humana, Inc., were extracted from the database of the company providing diagnostic imaging prior authorization to the health plans. Although the health plans were offered nationally, their membership was concentrated in the South. All the health plans were subject to the same prior authorization program for diagnostic imaging, as the insurer contracted with only one diagnostic imaging prior authorization company.

The prior authorization company implemented a nondenial approach to prior authorization, in which ordering physicians were required to engage in a discussion with a peer physician if their orders did not appear to be indicated, but always received authorization, regardless of the outcome of the conversation. [13] The Current Procedural Terminology (CPT) codes used for the extraction are listed in Table 1. The study received Institutional Review Board (IRB) approval from Advarra, with IRB Number Pro00027576.

**Table 1:** Current Procedure Terminology (CPT) codes used to define groups in the study.

Anatomy	Use of Contrast	CPT Codes
Abdomen	With	74160, 74177
Abdomen	Without	74150, 74176
Abdomen	Combined	74170, 74178
Thorax	With	71260
Thorax	Without	71250
Thorax	Combined	71270
Brain	Any	70450, 70460, 70470
Sinus	Any	70486, 70487, 70488

### Measurement

Two different grouping methodologies were used to calculate combined order rates pertaining to three different anatomical areas. When physicians ordered CT imaging, they contacted a prior authorization company to obtain authorization to do so. Each order designated both the facility of the physician requesting the order (the “ordering facility”), and the facility at which it was requested for the imaging to be performed (the “rendering facility”). The analysis was first conducted by grouping imaging orders according to the ordering facility, and then was conducted a second time by grouping orders according to the rendering facility. While ordering facilities can control the rates at which they order combined imaging, rendering facilities are unable to control the rate at which physicians (in some cases, based at other facilities) request that they perform combined imaging. As any facility may order a CT, but a facility must possess a CT machine in order to render a CT, there are far more ordering facilities than rendering facilities.

The three anatomical areas considered were the abdomen, thorax, and brain/sinus. The abdominal CT combined imaging rate of a facility was determined by dividing its number of combined orders for abdominal CT imaging by its total number of orders for abdominal imaging. The same process was used to determine the thoracic CT combined imaging rate. In contrast, the combined imaging rate for brain/sinus was calculated by dividing the number of brain CT orders paired with a sinus CT order for the same patient, placed on the same day, at the same facility, by the overall number of brain CT orders for the facility. Each time rates were calculated, facilities were excluded if they had ten or fewer orders in order to protect patient privacy and only consider facilities with meaningful volumes of orders. As orders containing clinically-warranted combined imaging were not excluded, it is not possible to compare the results with CMS HOQRP measures, which implement these exclusions. Thus, the rates reflect the actual unadjusted rates of combined imaging.

### Outcomes and analysis

Descriptive statistics for the rates across facilities were calculated. Descriptive statistics reported include the mean, median, interquartile range, standard deviation, and skewness in order to capture the distribution of outcomes. One-way analysis of variance was used to determine whether there were significant differences in the rates by year, facility type (hospital versus non-hospital), urbanicity, plan type (commercial versus Medicare Advantage), or region (NonSouth versus South). Rural areas were defined using a ZIP Code mapping created by the Centers for Medicare & Medicaid Services. [14] For the regional analysis, states were attributed to the South in accordance with the definition used by the U.S. Census Bureau. [15] The South consisted of the following (by postal abbreviation): AL, AR, DE, DC, FL, GA, KY, LA, MD, MS, NC, OK, SC, TN, TX, VA, and WV. This regional categorization was used because in each of the analyses, the majority of the orders were made by Southern ordering facilities and designated Southern rendering facilities.

## Results

**Fig 1:**
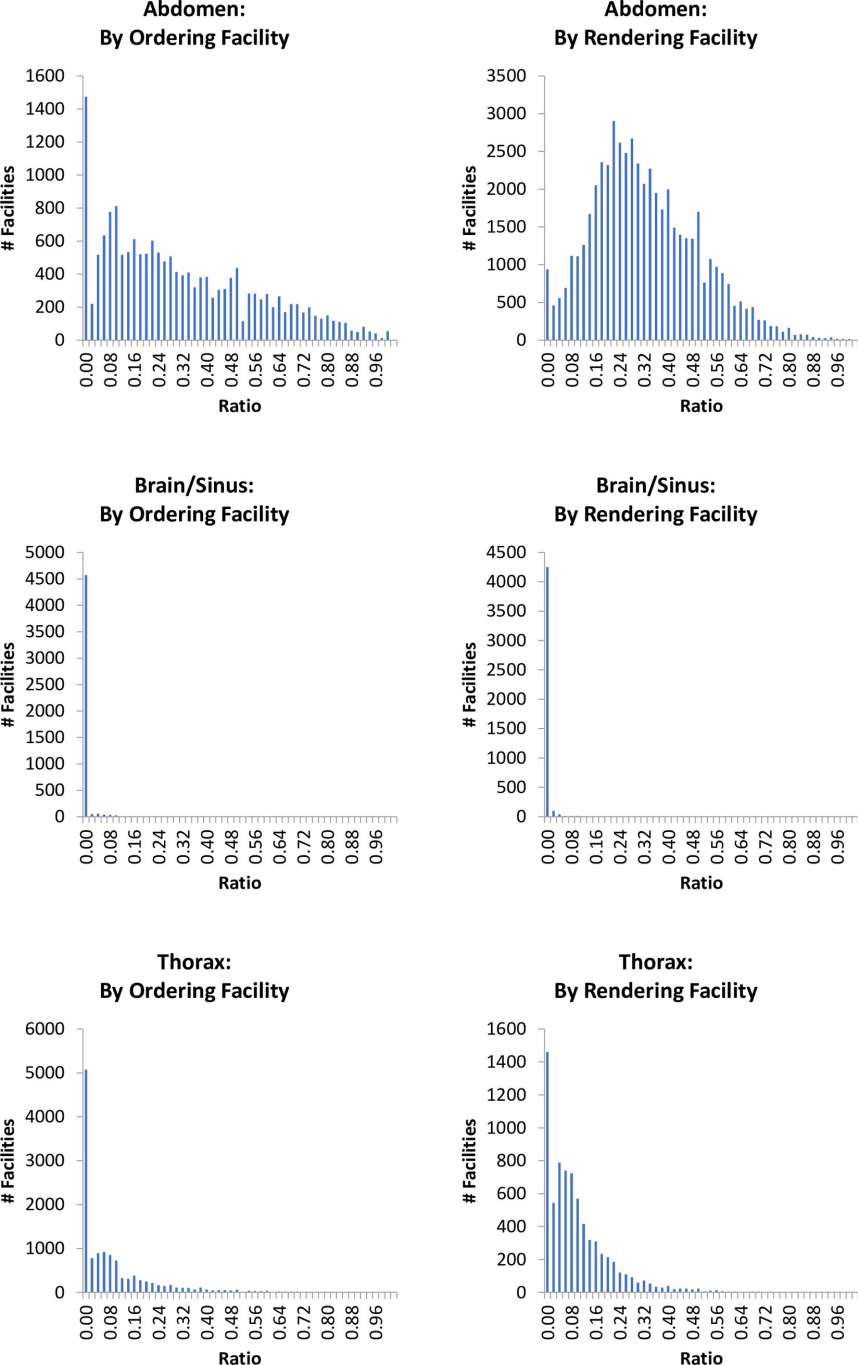
Histograms of combined imaging rates, by facility, 2013 to 2017.

Facility characteristics and descriptive statistics for the rates of combined imaging by facility are provided in Table 2, and histograms depicting the distributions of the combined imaging rates are shown in Fig 1. Combined imaging was ordered at a mean rate of 0.306, 0.089, and 0.002, for abdominal, thoracic, and brain/sinus imaging respectively. Combined imaging likewise was rendered at a mean rate of 0.311, 0.096, and 0.001, for abdominal, thoracic, and brain/sinus imaging respectively. The distribution of the combined imaging rate for abdominal CT followed a bell shape for rendering facilities (IQR: 0.182–0.424), and had a wider bell shape (IQR: 0.093–0.476), with a spike at zero for ordering facilities. In contrast, the vast majority of ordering and rendering facilities had no combined brain/sinus imaging. The distribution of combined thoracic imaging by ordering and rendering facility had an asymptotic distribution. The degree of variation, as represented both by the standard deviations of the combined imaging rates and the widths of their interquartile ranges, was greatest for abdominal CT, and smallest was for brain/sinus CT.

**Table 2:** Descriptive statistics for combined imaging rates across facilities, 2013 to 2017.

	Abdomen – By Ordering Facility	Abdomen – By Rendering Facility	Thorax – By Ordering Facility	Thorax – By Rendering Facility	Brain/Sinus– By Ordering Facility	Brain/Sinus– By Rendering Facility
# Orders	1,052,294	1,052,294	877,761	877,761	247,527	247,527
―% Commercial	20.0%	20.0%	13.1%	13.1%	14.7%	14.7%
# Facilities	16,972	8,203	12,630	7,306	4,779	4,410
―% Hospital	7.4%	56.1%	9.5%	57.4%	7.4%	61.7%
―% Rural	17.8%	21.5%	16.6%	20.9%	18.4%	23.2%
―% NonSouth	35.7%	45.1%	38.5%	43.5%	29.7%	38.7%
25^th^ Percentile	0.093	0.182	0.000	0.016	0.000	0.000
50^th^ Percentile (Median)	0.250	0.286	0.030	0.063	0.000	0.000
75^th^ Percentile	0.476	0.424	0.111	0.134	0.000	0.000
Mean Rate	0.306	0.311	0.089	0.096	0.002	0.001
―Standard Deviation	0.246	0.178	0.142	0.113	0.01	0.006
Skewness	0.684	0.615	2.575	2.267	6.879	10.144

As is shown in Table 3, combined imaging rates for abdominal CT and thoracic CT orders, grouped by both ordering and rendering facility, decreased monotonically from 2013 to 2017. In all four cases, the change over time was significant (*P* < .001). While the extremely low rates of combined brain/sinus imaging differed significantly by year when grouped by ordering facility (*P* < .01), there was not a monotonic trend. Rates of combined brain/sinus imaging did not differ significantly over time when orders were grouped by rendering facility (*P* = .21).

**Table 3:** Stratified combined imaging rates.

	Abdomen – By Ordering Facility	Abdomen – By Rendering Facility	Thorax – By Ordering Facility	Thorax – By Rendering Facility	Brain/Sinus– By Ordering Facility	Brain/Sinus– By Rendering Facility
**Year (P-Value)**	<0.001	<0.001	<0.001	<0.001	<0.01	0.206
―2013	0.347	0.356	0.091	0.125	0.000	0.001
―2014	0.329	0.333	0.072	0.102	0.001	0.001
―2015	0.306	0.310	0.061	0.088	0.002	0.001
―2016	0.287	0.293	0.047	0.072	0.001	0.001
―2017	0.272	0.276	0.038	0.060	0.002	0.001
**Site (P-Value)**	<0.001	<0.001	<0.01	0.879	0.285	0.346
―Hospital	0.260	0.292	0.078	0.096	0.003	0.001
―Non-Hospital	0.309	0.334	0.090	0.096	0.002	0.001
**Urbanicity (P-Value)**	0.005	<0.001	<0.001	<0.001	0.244	0.858
―Rural	0.294	0.289	0.100	0.107	0.002	0.001
―Urban	0.308	0.316	0.086	0.093	0.002	0.001
**Plan Type (P-Value)**	<0.001	<0.001	0.025	0.049	0.448	0.115
―Commercial	0.316	0.325	0.077	0.099	0.002	0.001
―Medicare Advantage	0.300	0.308	0.084	0.093	0.002	0.001
**Region (P-Value)**	<0.001	<0.001	<0.001	<0.001	0.500	0.516
―NonSouth	0.273	0.282	0.074	0.084	0.002	0.001
―South	0.324	0.333	0.098	0.105	0.002	0.001

Combined abdominal CT orders happened at a significantly lower rate at hospitals than at non-hospitals, both when the analysis was conducted according to ordering (.260 vs. .309; P < .001) and rendering (.292 vs. .334; *P* < .001) facility. Combined thoracic CT orders likewise constituted a significantly (.078 vs. .090; *P* < .01) smaller portion of orders when a hospital was designated as the ordering facility. No evidence was found to support the existence of a relationship between the nature of the site of service and the rate of combined brain/sinus CT.

Rural facilities had a significantly smaller rate of combined abdominal CT orders both when the analysis was grouped by ordering (*P* = .005) and rendering (*P* < .001) facility. Nonetheless, the difference was small in absolute magnitude; 0.294 for rural versus 0.308 for urban when the analysis was grouped according to ordering facility, and 0.289 for rural versus 0.316 for urban when the analysis was grouped according to rendering facility. However, rural facilities had a greater rate of combined thoracic CT orders than urban facilities, regardless of whether they were the ordering (0.100 versus 0.086; *P* < .001) or rendering (0.107 versus 0.093; *P* < .001) facility.

Combined abdominal CT rates pertaining to people with commercial health plans were significantly higher than rates pertaining to people with Medicare Advantage health plans when orders were grouped by ordering facility (0.316 versus 0.300; *P* < .001) and rendering facility (0.325 versus 0.308; *P* < .001). Combined thoracic CT rates pertaining to people with commercial health plans were significantly lower than rates pertaining to people with Medicare Advantage health plans when orders were grouped by ordering facility (0.077 versus 0.084; *P* = .025), but were significantly higher when orders were grouped by rendering facility (0.099 versus 0.093; *P* = 0.049).

Southern facilities were significantly more likely to order (0.324 versus 0.273; *P* < .001) and to be designated as the rendering facility for (0.333 versus 0.282; *P* < .001) combined abdominal CT. Likewise, Southern facilities were more likely to order (0.098 versus 0.074; *P* < .001) and to be designated as the rendering facility (0.105 versus 0.084; *P* < .001) for combined thoracic CT.

## Discussion

This study presents the first ever analysis of combined CT ordering and rendering in the context of a population with health plans from a source other than Traditional Medicare. It additionally presents the first ever analysis of combined CT ordering behavior. The findings showed significant differences between orders pertaining to commercial and Medicare Advantage populations, suggesting that health plan type may impact ordering behaviors.

There was not a uniform trend regarding the impact of health plan type; when orders were grouped by ordering facility, combined abdominal CT rates were significantly higher for orders pertaining to patients with commercial health plans, and combined thoracic CT rates were significantly lower for orders pertaining to patients with commercial health plans. As Medicare Advantage plans are primarily held by people aged 65 and older, there may be clinical differences between the two populations which led to this finding.

All the orders included in the analysis were authorized by a non-denial prior authorization program with a quality improvement focus. While all CT orders were subject to this process, combined brain/sinus orders have received particular attention from the program. A prior study reported that from 2010 to 2014, the prior authorization program received 113 requests for a combination of a head and a sinus/skullbase CT, leading to 64 collaborative consultations, and 19 instances in which ordering physicians agreed to drop one portion of the order. [16] Thus, the active efforts of the program to reduce unnecessary imaging orders are reflected in the decreasing combined order rates presented in the findings.

The trend of decreasing combined imaging order rates is consistent with trends seen in claims from the Traditional Medicare population. Analyses of Traditional Medicare claims data, which have excluded patients with clinically indicated combined imaging (unlike this analysis) and only incorporated data pertaining to hospital-based outpatient imaging, have found decreasing trends in combined imaging claims rates for abdominal and thoracic CT, but not for brain/sinus CT. [3, 8, 10, 11, 17] A study comparing HOQRP measures from 2011 with 2016 observed decreases in all three measures. [4] Thus, the trends found in the Traditional Medicare analyses somewhat mirror the patterns found in this analysis.

Several factors may have led to the decrease in combined imaging observed by this and other studies. The increased adoption of dual-energy CT systems capable of producing virtual unenhanced images is a one factor that may have contributed to some of the reduction in combined imaging, as this technology reduces the need for capturing a pre-contrast image. [18] Another factor may have been the increased attention that combined imaging has received as a result of the implementation of the HOQRP measures and the publication of measure performance on the Hospital Compare website. The HOQRP may have made ordering physicians more conscious of their use of combined imaging, as well as the need to use it judiciously. It is likewise possible that efforts to reduce combined imaging by private health plans may have had a spillover onto Traditional Medicare, as most physicians treat patients with both private health plans and Traditional Medicare. Further research is needed to better understand the factors that have led to the decrease.

Combined abdominal and thoracic imaging were significantly less common in hospitals than in other settings, except when thoracic orders were analyzed by rendering facility. As HOQRP quality measures [3, 8, 17] have examined combined imaging only in a hospital setting, if nonhospital care were impacted, it may have been due to a spillover effect. The lower rates of combined ordering seen in hospitals may be a product of the sentinel effect; higher quality of care is provided by physicians that are aware that they are being carefully watched. [19]

The regional differences observed were largely directionally similar to those observed in an analysis of 2014 Traditional Medicare claims. [12] The prior study found that the South had rates of combined abdominal CT and combined thoracic CT which were higher than the national average. Nonetheless, the prior study observed higher rates of combined abdominal and thoracic CT in rural areas, whereas this study observed lower rates of combined abdominal CT but higher rates of combined thoracic CT in rural areas.

### Limitations

There are a number of limitations that may have impacted the findings of this study. Although facilities with ten or fewer orders were excluded from the analyses, many facilities nonetheless had relatively low volumes of orders. The law of large numbers suggests that these low volume facilities are less likely to report rates representative of their true expected value than are high volume facilities; the rates low volume facilities report are more influenced by chance. There was a trade-off in reporting stable averages versus reporting findings including lower volume facilities, and the cut-off of a minimum of eleven orders was used to strike the balance.

While CMS reports outpatient combined imaging rates related to abdominal CT (OP-10), thoracic CT (OP-11), and brain/sinus CT (OP-14) through the HOQRP, it is not possible to draw a comparison between the rates reported by CMS and by this study due to a series of methodological differences. The CMS rates are based upon Traditional Medicare claims pertaining to combined CT performed in a hospital-based outpatient setting, and only examine claims for patients where combined imaging would be inappropriate. Patients with conditions warranting combined imaging are excluded from the numerator and denominator of the rates calculated by CMS. In contrast, the rates reported in this study pertain to Medicare Advantage orders occurring in all outpatient settings. No patients were excluded on the basis of appropriate combined imaging; thus, the rates reported by this study are higher than those reported by CMS. As was mentioned in the Introduction, there are a number of clinical situations in which combined abdominal, thoracic, and brain/sinus CT are clinically appropriate. The inclusion of patients with appropriate combined imaging may have influenced the differences observed in the stratified analyses, as there is no way of knowing whether observed differences were due to differences in the proportion of patients with appropriate indications.

The rates reported by this study may not be generalizable to other populations. The data analyzed pertain to health plans from one national insurer. Orders were authorized by one prior authorization company, and the rates of combined imaging were potentially influenced by its practices, which may differ from those of other prior authorization companies. While the combined imaging rates calculated for each facility included orders pertaining to patients belonging to health plans from one national insurer, the facilities likely provided services to patients affiliated with other insurers. It is unknown whether the facility-specific rates of combined orders calculated within this study are consistent with the rates of combined orders for patients affiliated with other insurers.

Lastly, as orders were analyzed, rather than claims, the extent to which combined imaging was conducted is unknown. Orders may not lead to claims if patients do not pursue imaging. This may happen for a variety of reasons, including change in clinical status, death, or various other patient concerns. Furthermore, the imaging performed may not match the imaging authorized during the ordering process, although such a mismatch could potentially lead to the claim’s payment being denied. Likewise, patients could potentially receive imaging at a facility other than the rendering facility named on their order. Despite these limitations, the orders analyzed validly represent the intentions of the ordering physicians after completing the prior authorization process.

## Conclusions

Significant differences in the rates of combined CT orders were observed between the commercial and Medicare Advantage populations, both when orders were grouped by ordering and designated rendering facility. Although the findings cannot be directly compared to prior research on the Traditional Medicare population, they suggest that health plan type may be a factor in the ordering and rendering of combined CT. The facility at which a patient receives care may also impact whether or not the patient has combined imaging. As such, there may be an opportunity to decrease variation between facilities in ordering and rendering combined imaging, to the extent that such variation is driven by factors other than clinical necessity.
